# Depletion of CD4 and CD8 T Cells Reduces Acute Disease and Is Not Associated with Hearing Loss in ML29-Infected STAT1-/- Mice

**DOI:** 10.3390/biomedicines10102433

**Published:** 2022-09-29

**Authors:** Rachel A. Reyna, Junki Maruyama, Emily K. Mantlo, John T. Manning, Satoshi Taniguchi, Tomoko Makishima, Igor S. Lukashevich, Slobodan Paessler

**Affiliations:** 1 Department of Pathology, University of Texas Medical Branch, Galveston, TX 77555, USA; 2 Department of Microbiology and Immunology, University of Texas Medical Branch, Galveston, TX 77555, USA; 3 Department of Otolaryngology, University of Texas Medical Branch, Galveston, TX 77555, USA; 4 Department of Pharmacology and Toxicology, University of Louisville, Louisville, KY 40292, USA

**Keywords:** Lassa virus, Lassa fever, arenavirus, ML29, sensorineural hearing loss model, STAT1-/- mice

## Abstract

Lassa virus (LASV) is a zoonotic virus endemic to western Africa that can cause a potentially lethal and hemorrhagic disease, Lassa fever (LF). Survivors suffer a myriad of sequelae, most notably sudden onset sensorineural hearing loss (SNHL), the mechanism of which remains unclear. Unfortunately, studies aiming to identify the mechanism of these sequelae are limited due to the biosafety level 4 (BSL4) requirements of LASV itself. ML29, a reassortant virus proposed as an experimental vaccine candidate against LASV, is potentially an ideal surrogate model of LF in STAT1-/- mice due to similar phenotype in these animals. We intended to better characterize ML29 pathogenesis and potential sequelae in this animal model. Our results indicate that while both CD4 and CD8 T cells are responsible for acute disease in ML29 infection, ML29 induces significant hearing loss in a mechanism independent of either CD4 or CD8 T cells. We believe that this model could provide valuable information for viral-associated hearing loss in general.

## 1. Introduction

Lassa virus (LASV) is endemic throughout western Africa and causes a potentially hemorrhagic disease, Lassa fever (LF) [1,2]. The virus, a member of the genus *Mammarenavirus* and family *Arenaviridae*, is zoonotic and spreads to humans through exposure to infected excretions or secretions of the *Mastomys natalensis* rodent, although recent studies indicate the rodent species *M. erythroleucus* and *Hylomyscus pamfi* may also serve as natural reservoirs [1,2,3]. The Centers for Disease Control and Prevention (CDC) estimates up to 300,000 new infections each year occur in West Africa, with as many as 10,000 deaths from LF [2,4]. While hospitalized cases have a case fatality rate of 15–20%, the overall case fatality rate is around 1% [2]. Around 80% of LASV-infected cases are asymptomatic, or present with a mild and vague febrile illness, which contributes to the high rate of misdiagnosis [1,2,5,6]. The other 20% of cases progress to develop much more severe symptomology, including hemorrhagic and neurologic disease [2]. There are no FDA-approved vaccines or therapeutics for LF, although an off-label use of ribavirin may be effective if administered early in disease [2,7]. 

LF survivors often suffer from life-long sequelae, including sudden-onset sensorineural hearing loss (SNHL), ataxia/imbalance, encephalopathy, and vision impairment [8,9,10]. Sudden-onset SNHL is the most common, with one-third of survivors developing this life-altering sequela [4,10,11]. Two-thirds of these cases are permanent and there is no way to predict when or how the hearing loss will present; onset can be unilateral or bilateral and can occur during both the acute and convalescent phases of infection in both clinical and subclinical cases [11]. Ribavirin treatment cannot slow or prevent onset of SNHL induced by LASV infection [11]. Neurological sequelae, such as SNHL, generate a huge socioeconomic burden on affected countries [7]. Nigeria alone spends upwards of USD 43 million each year for aid programs [7]. Patients with sequelae often suffer isolation and stigmatization, as well as increased rates of depression and unemployment [12]. 

Understanding the mechanisms driving sequelae is important to better develop therapeutics and interventions for LF survivors. Current research into these mechanisms implicate the involvement of the immune response, notably the LASV-specific T cells, in the protection against disease and the development of SNHL. While an early and robust T cell response against LASV is known to be important for both protection against and recovery from LF in both humans and NHPs [13,14,15], these LASV-specific T cells have also been implicated in acute disease as well as sequela in several experimental models [16,17,18,19]. Previous work with nonhuman primates demonstrates that LF survivors may develop hearing loss, as measured by the Brainstem Auditory Evoked Response [16]. Currently, there is a reliable murine model for SNHL; STAT1-/- mice surviving LASV infection develop hearing loss at a high rate, as measured by the acoustic startle response [17]. Furthermore, we recently published that more clinically relevant testing methods, auditory brainstem response (ABR) and distortion product otoacoustic emission (DPOAE) testing, are able to detect hearing loss in STAT1-/- mice surviving LASV infection and that this hearing loss is likely CD4 T cell-mediated [19]. These studies suggest the involvement of the LASV-specific immune response in the development of this particular sequela. Unfortunately, all studies using LASV are limited due to biosafety level 4 (BSL-4) facility constraints and therefore animal models for lower containment are needed to further investigate immune and pathological mechanisms resulting in sequelae.

Thus, a surrogate model mimicking development of LASV-specific host responses is desirable to reveal a more detailed mechanism needed for targeted development of countermeasures. An ideal surrogate model would be able to be used in a low-containment setting, produce a similar clinical disease, and ultimately develop signs of desired sequelae. One such potential surrogate candidate is the highly attenuated ML29. ML29 is a reassortant, biological clone that consists of the S segment of LASV Josiah and the L segment of the non-human pathogenic Mopeia virus (MOPV), isolated after a co-infection of cells with both viruses [20,21]. In the S segment, ML29 encodes the major antigens of LASV, the glycoprotein precursor (GPC) and the nucleoprotein (NP) [20]. In addition, ML29 contains eighteen clone-specific mutations that distinguish its genome from parental viruses and are likely contributing to its attenuated phenotype [20]. Based on ML29 genetic sequence, the GPC and NP should trigger a similar immune response as LASV, and thus allow one to study all beneficial and negative consequences of that response in animal models. Previous studies with ML29 have confirmed its reproducible virulence in STAT1-/- mice [22]. Moreover, ML29 is not a select agent and can be used safely in BSL-2 facilities, making it accessible to a wider research field. This study examines the application of ML29 as a surrogate model for LF. Here, we used immunocompromised STAT1-/- mice experimentally infected with recombinant ML29 (rescued from cDNA) to study the contributions of the T cells response to acute disease and sequelae. We confirmed the pathogenicity of recombinant ML29 in STAT1-/- mice and demonstrate the involvement of both CD4 and CD8 T cells in the acute phase of disease. However, hearing loss detectable in this model appears to occur through a T cell-independent mechanism and is therefore likely different than results reported previously for LASV infection.

## 2. Materials and Methods

### 2.1. Animal Experiments

Four-to-eight-week-old STAT1-/- mice or their parental wild-type (WT) 129S6/SvEv were purchased from Taconic Biosciences, Inc. for each experiment. All mice were subcutaneously implanted with a BMDS IPTT-300 transponder encoding mouse identification and allowing for the measurement of body temperature via a DAS-8007 transponder reader (Bio Medic Data Systems, Seaford, DE, USA). Mice were intraperitoneally injected with either sterile PBS or up to 10^5^ PFU of ML29 in 100 μL of sterile PBS. Mice that were depleted of T cells received 100 μg of InVivoPlus anti-mouse CD4 (GK1.5, BioXCell, Lebanon, NH, USA) and/or anti-mouse CD8a monoclonal antibodies (mAb) (53–6.7, BioXCell) intraperitoneally three and one day prior to infection as previously described [19]. Mock depletions were achieved using InVivoPlus rat IgG2b isotype control and/or InVivoPlus rat IgG2a isotype control (BioXCell). 

All mice were monitored daily for signs of disease, with body weights recorded daily at least until 28 days post-infection or post-splenocyte transfer. Mice that reached humane endpoint criteria (≥25% weight loss, body temperature < 32 °C, or an inability to access food or water) or the scientific endpoint were humanely euthanized using CO_2_ asphyxiation followed by cervical dislocation. All animal protocols were approved by the Institutional Animal Care and Use Committee at the University of Texas Medical Branch and are compliant with National Institutes of Health guidelines. 

### 2.2. Auditory Function Analysis

Auditory tests were performed once prior to the beginning of each study to set a baseline, and then every other week beginning as early as three weeks post-infection. Mice were first anesthetized with isoflurane, then intraperitoneally injected with a mixture of 100 mg/kg of ketamine with 10 mg/kg of xylazine in PBS. Testing commenced once the mouse became unresponsive to a tail or foot pinch. All auditory tests were performed within a soundproof box.

The Auditory Brainstem Response (ABR) analysis was conducted using the Intelligent Hearing System SmartEP software as previously described [19,23]. In total, 256 responses elicited by a click stimulus (broad band frequency, peak range 0.5–16 kHz) were averaged and recorded in a series of descending 5–10 decibel (dB) steps. The auditory threshold was determined by identifying the smallest decibel stimulus that elicited recognizable waveforms.

The Distortion Product Otoacoustic Emission (DPOAE) analysis was conducted using the Starkey DP2000 system (Starkey Laboratories, Eden Prairie, MN, USA), as previously described [19,23]. A modified ear probe, adjusted to fit the mouse auditory canal, was used. The distortion product 2F_1_-F_2_ was obtained using the high frequency setting, with the F_2_ values ranging between 8–16 kHz. The sum of all DP/NF values across all tested F_2_ frequencies was used for comparison between mice. All negative DP/NF values were corrected to 0 for calculations.

ABR and DPOAE testing was performed in the right and left ear individually using ear inserts for all mice and data from each ear were used for analyzing test results. If abnormal results were obtained from an ear upon baseline testing, data from that ear were not used for the study. If abnormal results were obtained from both ears, the mouse was excluded from data analysis. 

### 2.3. Cells and Virus

Vero cells were maintained in Dulbecco’s modified Eagle’s medium (DMEM) supplemented with 10% fetal bovine serum (FBS), 1% penicillin-streptomycin, and L-glutamine. Recombinant ML29 was provided by Dr. Igor S. Lukashevich (University of Louisville, Louisville, KY, USA). Recombinant ML29 was rescued from cDNA clones as previously described [24] and was propagated in Vero cells. Virus-containing cell culture supernatant was stored at −80 °C until use. All infectious work was performed in biosafety level 2 (BSL-2) facilities in the Galveston National Laboratory at the University of Texas Medical Branch (UTMB) in accordance with institutional guidelines.

### 2.4. Viral Titration

All viral titers were determined using plaque assays. Confluent monolayers of Vero cells were prepared on 12-well plates the day prior to infection in DMEM supplemented with 10% FBS, 1% penicillin-streptomycin, and L-glutamine. Cells were inoculated with 100 μL of 10-fold serially diluted virus and incubated for 45 min at 37 °C in a CO_2_ incubator with 5% CO_2_. After removing the inoculum, each well was overlaid with minimum essential medium (MEM) containing a final concentration of 2% FBS, 1% penicillin-streptomycin, and 0.6% tragacanth (Sigma, Burlington, MA, USA). Cells were fixed with 10% formalin and stained with crystal violet after a 7-day incubation. Viral titers were represented as plaque forming units (PFU).

### 2.5. Histological Analysis

Temporal bones of mice were processed for paraffin embedded thin sections as described previously [19]. Briefly, the temporal bones were dissected, fixed for >72 h in 10% formaldehyde, decalcified in 0.1M EDTA for >7 days, and embedded into paraffin blocks using standard methods. The paraffin embedded inner ear tissue was then thin-sectioned at 5 µm onto slides and stained with hematoxylin and eosin. The slides were imaged using Leica DMLB microscope, Nikon Digital Sight 1000 digital camera and NIS Element F software (Nikon, Melville, NY, USA). 

### 2.6. Statistical Analysis

All statistics were performed using GraphPad Prism 9 software. Statistical significance was deemed with a *p* value < 0.05. Statistical significance for the organ samples from the double T cell depletion study was determined using an unpaired Student’s *t*-test. Significant differences for ABR and DPOAE testing was determined using a repeated measures two-way ANOVA followed by the Bonferroni multiple comparisons post hoc test. Differences between groups for the organ titrations were determined using a one-way ANOVA followed by Tukey’s multiple comparisons post hoc test. 

## 3. Results

### 3.1. Pathogenicity of ML29 in STAT1-/- Mice

**Figure 1:**
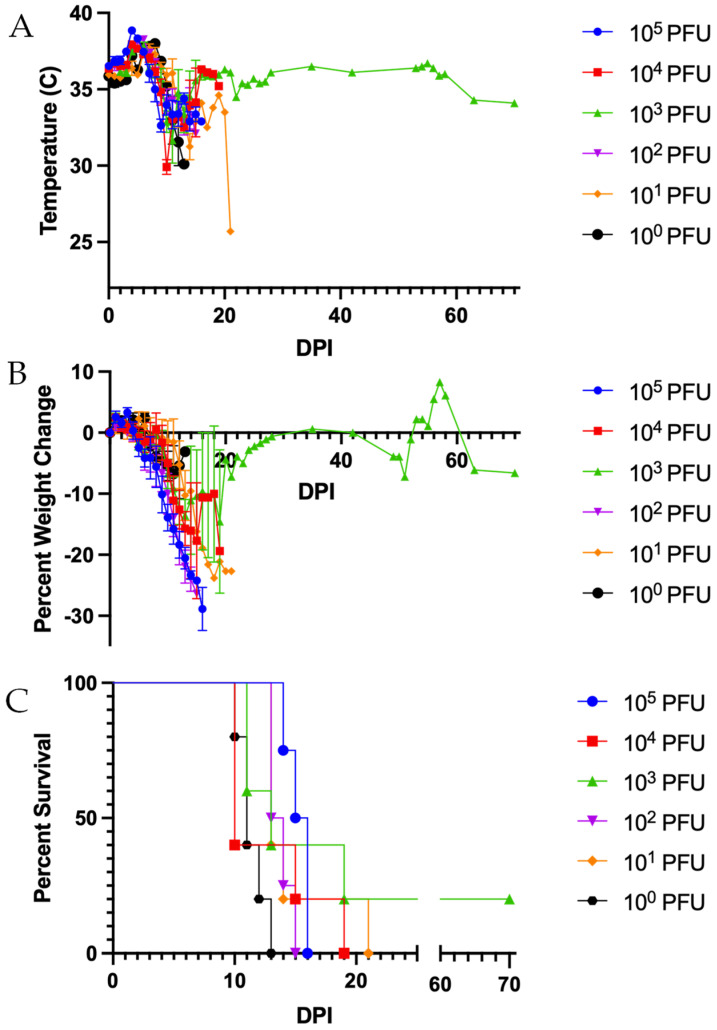
**ML29 causes a lethal infection in STAT1-/- mice with an LD50 of <1 PFU.** STAT1-/- mice were inoculated with 10^0^ to 10^5^ PFU of ML29. (**A**) Changes in body temperature post-infection. (**B**) Changes in body weight post-infection. (**C**) Percent survival: 10^4^, 10^3^, 10^1^, and 10^0^ PFU *n* = 5; 10^5^ and 10^2^ PFU *n* = 4. Data presented as average ± SEM for temperature and weight changes.

To determine the 50% lethal dose (LD_50_) of ML29, STAT1-/- mice were intraperitoneally inoculated with 10^0^ (*n* = 5), 10^1^ (*n* = 5), 10^2^ (*n* = 4), 10^3^ (*n* = 5), 10^4^ (*n* = 5), or 10^5^ (*n* = 4) PFU of ML29. All mice developed clinical signs of infection, including transient fever, hypothermia, and weight loss (Figure 1A,B). Other clinical signs included scruffy fur, hunched posture, and loose stool. All mice succumbed to infection in all groups by 21 days post-inoculation (dpi), except for one mouse from the 10^3^ PFU group (20% survival) (Figure 1C). The LD_50_ of ML29 was determined to be <1 PFU. No significant changes in survival were seen between groups. 

Virus was detected in the brain, lung, liver, spleen, kidney, and serum in at least one mouse from all groups at time of sampling (Table S1). Virus was completely cleared for all tested samples in only two mice: one mouse in the 10^4^ PFU group that succumbed on 19 dpi and the surviving mouse. 

As hearing tests cannot be performed until the mouse recovers from acute infection, ABR and DPOAE analyses were only performed on the sole survivor from the 10^3^ PFU group. While this mouse did not develop measurable hearing loss through 10 weeks post-inoculation (wpi) (Supplemental Figure S1), it did develop transient signs of neurological symptoms, such as imbalance and a body tremor. 

### 3.2. Pathogenicity of ML29 in CD4/CD8 T Cell Double Depleted STAT1-/- Mice

**Figure 2:**
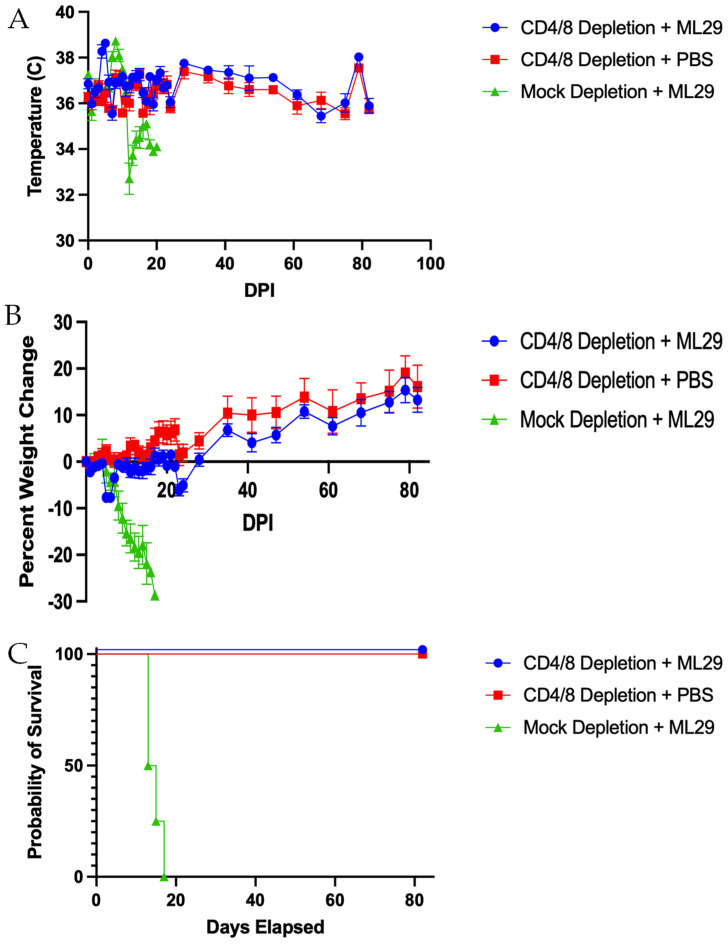
**Acute disease in ML29 infection is T cell dependent.** STAT1-/- mice were either mock depleted or depleted of both CD4 and CD8 T cells and inoculated with ML29 or PBS as a mock control. (**A**) Changes in body temperature post-infection. (**B**) Changes in body weight post-infection. (**C**) Percent survival. *n* = 5 Depletion + ML29; *n* = 4 Depletion + PBS; *n* = 4 Mock Depletion + ML29. Data presented as mean ± SEM for temperature and weight changes.

To assess the roles of T cells in ML29-induced pathogenicity for STAT1-/- mice, depletion of both CD4 and CD8 T cells or a mock depletion was performed, then mice were intraperitoneally inoculated with PBS or 10^3^ PFU of ML29. All infected mice in the mock-depleted group developed a transient fever followed by hypothermia and weight loss as expected (Figure 2A,B). In the CD4/CD8 T cell-depleted group, this weight loss was minor and transient, as compared to the mock-depleted group (Figure 2B). All mock-depleted mice succumbed to infection by 20 dpi, whereas all CD4/CD8 T cell-depleted mice survived infection (Figure 2C). Organ and serum samples collected at the study endpoint from survivors indicate systemic infection present through the end of the study (Supplemental Figure S2).

**Figure 3:**
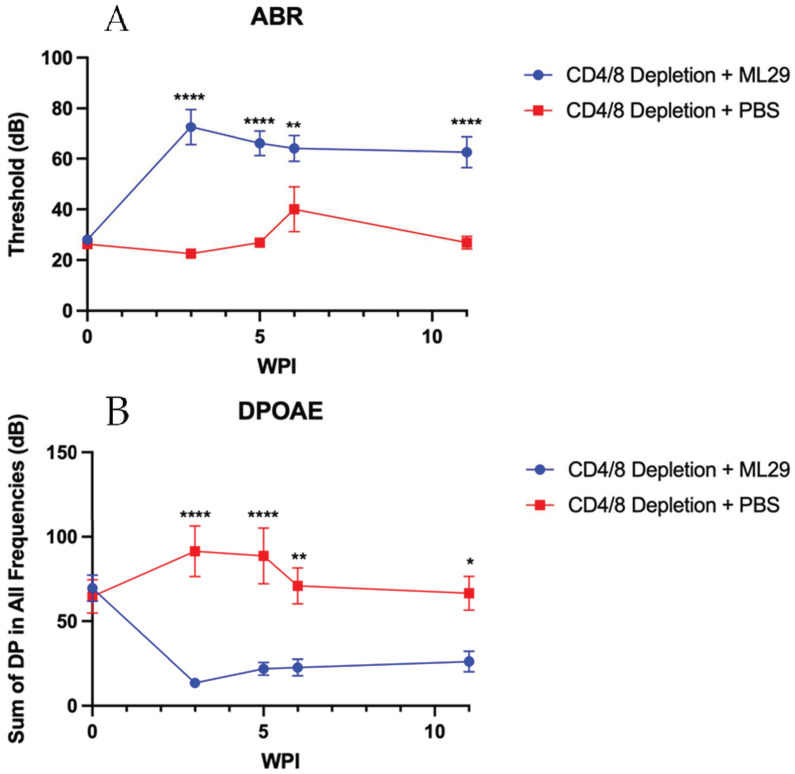
**Hearing loss occurs in a mechanism independent of CD4 and CD8 T cells during ML29 infection.** ABR (**A**) and DPOAE (**B**) were performed on the survivors of infection through 11 wpi. *n* = 10 Depletion + ML29; *n* = 8 Depletion + PBS. Data presented as mean ± SEM. * *p* < 0.05, ** *p* < 0.01, **** *p* < 0.0001.

ABR and DPOAE analysis were performed on survivors of infection, beginning at 3 wpi and continuing through 11 wpi. ABR analysis revealed that CD4/CD8 T cell-depleted mice surviving ML29 infection had a significant increase in their auditory threshold (Figure 3A). Similarly, DPOAE testing indicated these mice had a significant decrease in their distortion product (DP) value (Figure 3B). Both hearing tests indicate the development of significant hearing loss in these mice. No signs of imbalance were observed. 

**Figure 4:**
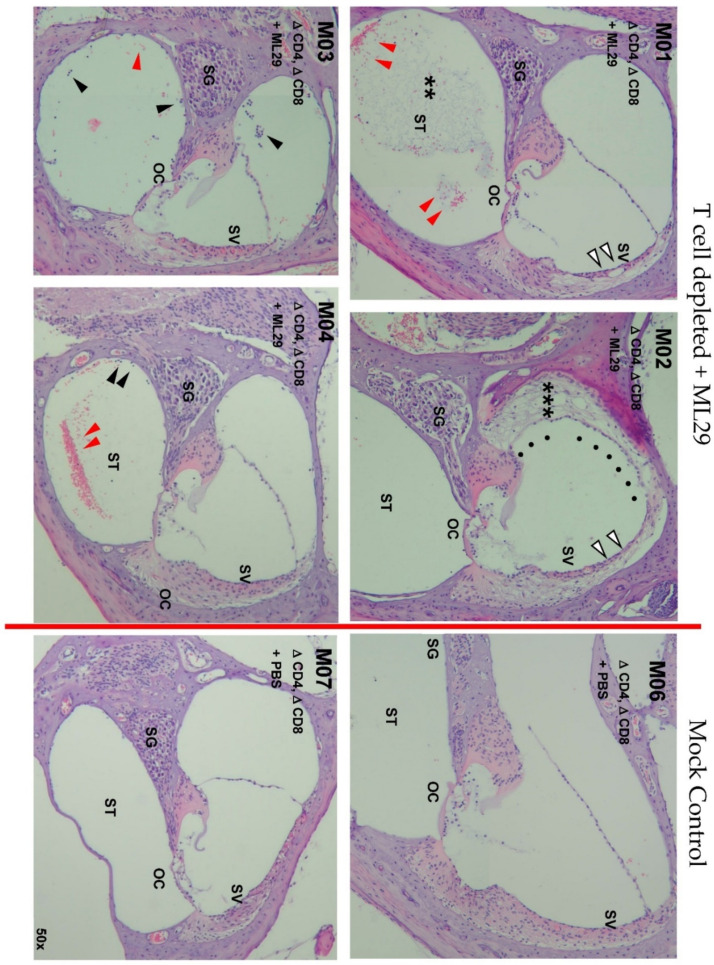
**Damage within the inner ear occurs in a CD4/CD8 T cell-independent mechanism.** Representative images of cross sections of the apical-middle turn of the cochlear duct are shown. PBS-inoculated mice have normal histology (M06 and M07). In all CD4/CD8 T cell-depleted, ML29-inoculated mice, the cochlear nerves remained mostly intact, although the nerves were surrounded by hemorrhage in many samples. Mild vacuolization is seen within the spiral ganglion (SG). Major thinning of the stria vascularis (SV) is seen (white arrowheads). The scala tympani (ST) features hemorrhage (red arrowheads) and white blood cell infiltration (black arrowheads). M01 presents with mild fibrous degeneration (**) within the ST, suggestive of inflammation. M02 presents with significant hydrops in the scala media as shown by the bowing of the Reissner’s membrane (black dots) and fibrous degeneration in within the perilymph in the scala vestibuli (***), indicative of severe inflammation. Minimal damage was seen in the hair cells and the organ of Corti (OC).

Histology of the inner ear demonstrated marked damage for the CD4/CD8 T cell-depleted mice infected with ML29 (Figure 4). While the cochlear nerves remained mostly intact, the nerves were surrounded by hemorrhage in many samples. Mild vacuolization within the spiral ganglion (SG), thinning of the stria vascularis (SV), hemorrhage and white blood cell infiltration in the scala tympani (ST) were some of the notable findings in the mice with hearing loss. M01 presents with mild fibrous degeneration within the ST, suggestive of inflammation. M02 presents with significant edema and fibrous degeneration in within the perilymph, bowing of the Reissner’s membrane, and hydrops in the scala media, indicative of severe inflammation. Minimal damage was seen in the hair cells and the organ of Corti (OC). All PBS-inoculated control mice had normal histopathology.

### 3.3. Pathogenicity of ML29 in Individual CD4 or CD8 T Cell Depleted STAT1-/- Mice

**Figure 5:**
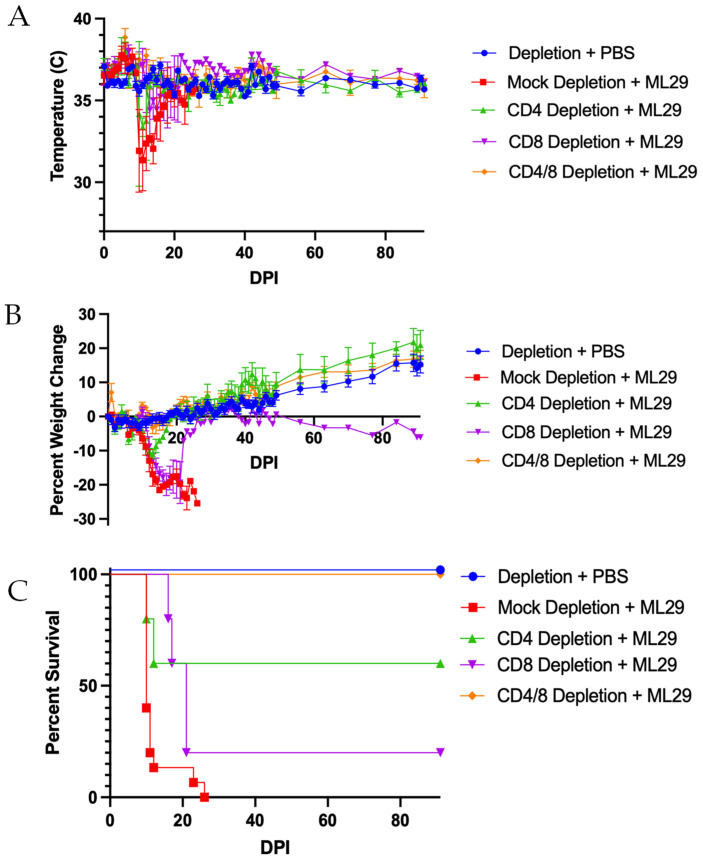
**Both CD4 and CD8 T cells are responsible for acute disease in ML29 infection.** STAT1-/- mice were depleted of either or both CD4 and CD8 T cells and inoculated with ML29 or PBS as a mock control. (**A**) Changes in body temperature post-infection. (**B**) Changes in body weight post-infection. (**C**) Percent survival. *n* = 10 Depletion + PBS; *n* = 15 Mock Depletion + ML29; *n* = 5 CD4 Depletion + ML29, CD8 Depletion + ML29 in panels (**B**,**C**), and CD4/8 Depletion + ML29; *n* = 4 CD4 Depletion ML29 in panel (**A**). Data.

STAT1-/- mice were depleted of either CD4, CD8, or both CD4/CD8 T cells and inoculated with either 10^3^ PFU of ML29 or PBS on D0. All mice that succumbed to infection followed the expected disease progression: transient fever, hypothermia, and severe weight loss (Figure 5A,B). In total, 100% of mock-depleted mice, 80% of CD8 T cell-depleted mice, and 40% of CD4 T cell-depleted mice succumbed to infection by 26 dpi (Figure 5C). All CD4/CD8 T cell-depleted mice survived infection. Serum and organ samples were collected at the time of euthanasia for virus titration. Several mice were in too poor condition for serum collection; organ samples were not collected from one mouse in the CD8 T cell-depleted, ML29-incoulated group. While the CD8 T cell-depleted mice succumbing to infection had a trend of lower organ and serum titers than the CD4 T cell-depleted counterparts, only the kidney showed significant differences between all three groups (Supplemental Figure S3).

**Figure 6:**
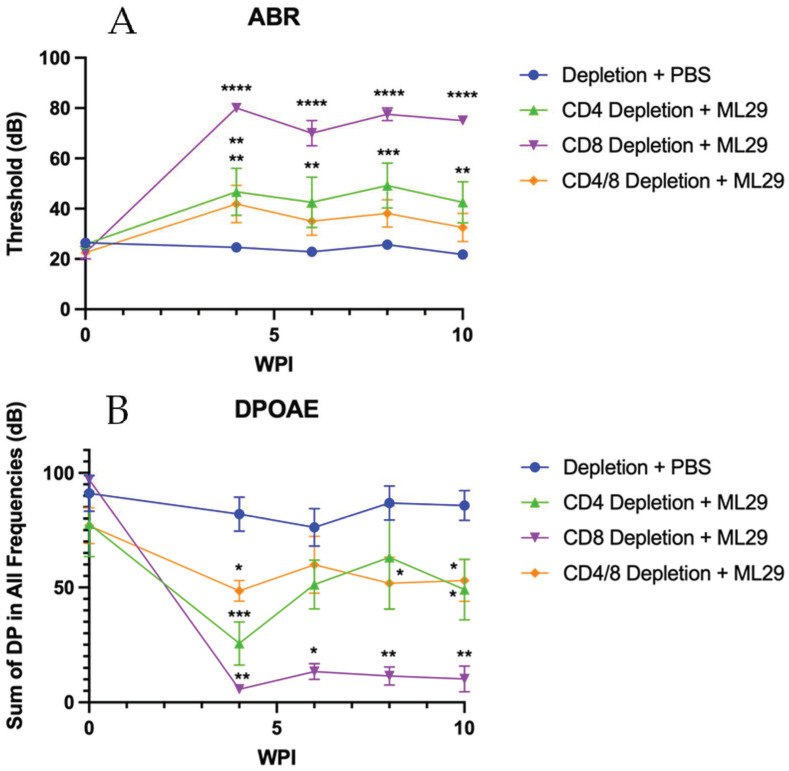
**ML29-inoculated mice appear to develop hearing loss through a T cell-independent mechanism.** ABR (**A**) and DPOAE (**B**) were performed on the survivors of individual CD4 or CD8 depletions. *n* = 14 Depletion + PBS; *n* = 8 CD4/8 Depletion + ML29; *n* = 6 CD4 Depletion + ML29; *n* = 2 CD8 Depletion + ML29. Data presented as mean ± SEM. * *p* < 0.05, ** *p* < 0.01, *** *p* < 0.001, **** *p* < 0.0001.

Survivors were monitored for the development of hearing loss through ABR and DPOAE analysis, beginning at 4 wpi and continuing through 10 wpi. CD4 and CD4/CD8 T cell-depleted mice demonstrated a mild to moderate hearing loss, as measured by ABR (Figure 6A) and DPOAE (Figure 6B). The sole CD8 T cell-depleted survivor featured more severe hearing loss, as measured by ABR and DPOAE.

## 4. Discussion

Survivors of LF often develop life-long sequelae, including sensorineural hearing loss, encephalopathy, ataxia, and vision impairment [8,9,10]. While the exact cause of these sequelae remains unknown, previous work using both murine and primate models implicates the involvement of the immune response [16,17,18,19]. Unfortunately, due to the constraints of working in BSL-4 facilities, a more thorough investigation of the exact mechanism behind these sequelae is difficult to perform, making a low containment surrogate model extremely desirable.

In immunocompetent mice, LASV and MOPV induce lymphocytic choriomeningitis virus (LCMV)-like immunopathology with outcomes dependent on H2 background, age of mice, and the dose/route of viral inoculation [25,26]. The ML29 clone was rationally selected from a library of MOPV/LASV reassortants to preserve the attenuated phenotype encoded by the MOPV L RNA segment and to induce LASV-specific protective immune responses as encoded by the LASV S RNA [21]. LASV-specific immune responses induced by ML29 in immunocompetent CBA/J mice were used as a vaccine potency tool to assess LASV-specific cytotoxic T lymphocytes and protection [27]. In this model, protection was fully dependent on CD8 T cells and partially on CD4 T cells. Recently, we have documented that CD8 T cells play a critical role in pathogenicity during the acute phase of LASV infection in STAT1-/- mice, while CD4 T cells play an important role in LASV-induced hearing loss during the chronic phase of infection [19].

The objective of this study was to determine whether the ML29 virus can serve as a suitable surrogate model for LASV by mirroring the immune contributions previously seen in murine LF in both acute disease and with the development of sequelae in STAT1-/- mice [19]. Our data indicate that recombinant ML29 is unsuitable to act as a surrogate model for sequelae testing using this immunocompromised STAT1/- model; the LD_50_ of <1 PFU results in no survivors to allow for convalescent phase testing. Moreover, testing cannot be performed during the acute phase of disease due to the poor health condition of infected mice. This difference may be due to the fact that this study was performed with recombinant ML29, while previous work [22] was performed using biological ML29. While both ML29 viruses are genetically identical and were plaque-purified to reduce quasi-species heterogeneity, we cannot exclude some differences between the two variants due to the presence of defective viral genomes (DVGs) and/or defective viral particles (DIPs). Generation of DVGs and DIPs during replication is a common feature of many viral infections, including mammarenaviruses [28,29,30,31]. DVGs and DIPs play a critical role in the modulation of viral load, innate immune responses, disease outcome, and viral persistence [28,29,30]. Due to the “inherited” mismatch between the L protein (MOPV) and NP protein (LASV), the ML29 polymerase complex generates abundant DVGs of the truncated L-derived RNA 1.5kb species [31]. The accumulation of these DVGs during the acute phase of ML29 infection may contribute to the high immunogenicity of ML29 and its attenuation in all tested animal models [32]. Indeed, previous work in STAT1-/- mice indicates that ML29 had a thousand-fold reduction in viremia and viral load in tissues in comparison to MOPV-infected animals [22].

Previous studies have implicated the immune response, most notably the T cell response, in animal models as a key player for both acute disease and sequelae [16,17,18,19]. Recently, an examination of the role of T cells in LASV-infected STAT1-/- mice indicated that CD8 T cells are responsible for acute disease while CD4 T cells may be a driving factor of SNHL [19]. In order be an acceptable surrogate model, we expected that ML29 infection of STAT1-/- mice would generate comparable results. Performing a series of T cell depletions, we confirm that T cells are important in development of the acute disease, although these contributions do not mirror what has been reported for LASV infection of STAT1-/- mice. While LASV acute disease appears to be driven primarily by CD8 T cells [19], both CD4 and CD8 T cells are responsible for acute disease in ML29 infection; 100% survival is only seen with a double depletion of both CD4 and CD8 T cells. CD4 T cells may play a stronger role in acute ML29-induced disease; CD8-depleted mice featured only a 20% survival, while CD4-depleted mice had 60% survival, although increased group sizes are required to validate this claim. Ultimately, the involvement of both CD4 and CD8 T cells for pathogenesis renders ML29 inadequate as an exact surrogate model for acute Lassa fever as LASV pathogenesis appears strictly CD8 T cell mediated [19].

Perhaps this difference in pathogenicity is due to the fact that MOPV is known to induce a stronger T cell response than LASV and has been previously demonstrated to be more lethal in STAT1-/- mice than ML29 and LASV [22,33,34,35,36,37,38]. Future work should examine the immune contributions of the MOPV RNA-dependent RNA polymerase (L) and the Really Interesting New Gene (RING) finger matrix (Z) proteins to ML29 pathogenesis and immunogenicity. The LASV Z protein is known to aid in the suppression of the innate immune response through direct interactions with retinoic acid-inducible gene I (RIG-I) [39]. Perhaps the MOPV Z protein is unable to suppress RIG-I as effectively, allowing for innate immune activation more readily. Moreover, the high rate of DVGs produced upon ML29 infection indicate an active MOPV L protein [31]. These truncated transcripts, if packaged and released from infected cells, would contribute to innate immune activation and ultimately the generated T cell response. Further work is required to better understand the contributions of LASV- and ML29-specific innate and adaptive immune responses to elucidate exact mechanisms behind ML29 pathogenesis.

As for sequelae, ML29 does not appear to behave similarly to LASV in this model as ABR or DPOAE results reported in this paper suggest. In our previous work, we found that LASV causes severe hearing loss in which both ABR and DPOAE tests are unable to detect any responses indicative of functional hearing [19]. CD4 T cell depletion is sufficient to prevent the development of hearing loss, indicating its role as the driving factor of SNHL [19]. However, the hearing impairment seen with ML29 is a moderate hearing loss, in contrast to the severe hearing loss seen in LASV infection; no matter the severity, both ABR and DPOAE were sensitive enough to detect significantly weakened hearing ability. This moderate hearing loss is present regardless of T cell depletion status, implying that ML29-mediated hearing loss occurs in a mechanism independent of either CD4 or CD8 T cells. Although further work is required to determine the exact mechanism of this hearing loss, a directly viral-mediated pathway cannot be rejected.

Histological analysis of the inner ears was performed for the preliminary CD4/CD8 T cell depletion study. All ML29-infected, T cell-depleted STAT1-/- mice demonstrated marked damage, with the severity of damage appearing to correlate with the severity of hearing loss. However, the histological damage seen in these samples is much less severe than that seen with LASV infection [19]. LASV infection results in increased damage to the spiral ganglion as well as the cochlear nerve. This emphasizes the CD4/CD8 T cell-independent mechanism; ML29 is highly immunogenic [20,22,27,40], and yet the inner ear damage and the associated hearing loss are not occurring with the same severity seen with LASV. These results further emphasize that ML29 and LASV-associated hearing loss are potentially occurring through two separate mechanisms.

When approaching vaccine development for a virus with such consistent, debilitating, and immune-mediated sequelae as LASV, maximum precautions must be taken to ensure there is no chance of triggering any form of disease or sequelae in vaccine recipients. Previous work has demonstrated ML29 to be clinically safe and immunogenic in immunocompetent models [20,27,40,41]. Taking into consideration the high prevalence of human immunodeficiency virus (HIV) infection in Nigeria and other areas of West Africa, ML29 has been tested in a simian immunodeficiency virus (SIV)-infected macaque model [42]. In this model, ML29 vaccination results in no clinical signs of disease, although a low and transient viremia was detected [42]. Notably, SIV-infected and ML29-vaccinated primates were fully protected against a fatal heterologous challenge with the WE strain of LCMV [32]. However, no studies have been conducted to thoroughly examine the possibility of sequelae in these immunocompromised models. Our sole survivor of ML29 infection presented with transient neurological signs. It has been recently documented that formulation of ML29 with DIPs resulted in complete attenuation of ML29 infection in STAT1-/- mice [22]. These mice featured transient weight loss and clinical manifestations during the first week of disease but recovered rapidly [22]. More importantly, these STAT1-/- mice inoculated with DIP-enriched ML29 did not exhibit signs of SNHL when assessed by the acoustic startle response throughout the entire observation period (62 days) [22], indicating these neurological signs may not be an adverse effect of ML29 vaccination.

ML29 virus infection and Lassa virus infection cause hearing loss in this particular model, although the mechanisms appear to differ [19]. One current hypothesis is that the CD4 T cells in the Lassa model could be dysregulated and/or frozen in a cytotoxic state and so contribute to neuronal damage in the auditory ganglion. This mechanism may open the possibility of using immunotherapies to treat LF patients developing SNHL. Dysregulation of T cells is an important topic in viral immunology as well as in oncology [43]. Previous research into the modulation of these T cells could provide insight for the eventual development of therapies to aid those suffering LF-associated SNHL. Although this ML29 model of infection would not be able to contribute to these studies, it is indeed a remarkable model for the study of viral-associated hearing loss. Current hearing loss models in mice often require significant surgical intervention or techniques that may not be readily accessible. For example, one extensively studied viral-associated hearing loss model focuses on cytomegalovirus (CMV). CMV causes congenital SNHL through a mechanism that is at least partially immune mediated [44,45,46]. Current models for CMV-associated hearing loss include both guinea pigs and mice [47,48,49,50,51,52]. While this model is readily accessible due to its BSL-2 classification, production of offspring with measurable hearing loss is not consistent when generated by infecting pregnant dams or by performing intrauterine infection [47,48,49,51]. Induction of consistent hearing loss and associated histopathological findings require the use of intracranial or intracochlear injections in pups [47,52], techniques that are difficult to perform. Other methods of generating hearing loss include a combination approach, by using an intraperitoneal infection with CMV followed by an intracranial injection of lipopolysaccharide [50]. In comparison, our ML29 murine model reliably develops viral-associated hearing loss in a simplistic manner; only a series of intraperitoneal injections are required, and more importantly, all mice develop measurable hearing loss. Further development of the ML29 model of hearing loss, perhaps through enrichment of ML29 inoculum with DIPs for further attenuation, will allow for the development of screening techniques and therapeutics.

In conclusion, our findings here demonstrate that both CD4 and CD8 T cells play a role in acute pathogenesis during ML29 infection in STAT1-/- mice; 100% survival is only achieved with a double CD4 and CD8 T cell depletion. Moreover, significant hearing loss occurs through a mechanism independent of CD4 and CD8 T cells. While future work is required to establish an exact mechanism, a hypothesis of direct viral contributions resulting in hearing loss cannot be eliminated. While our results indicate that ML29 is unlikely a suitable surrogate model to exactly mimic LF, this model can be helpful in the development and screening of potential therapeutics for viral-associated hearing loss.

## Data Availability

All data presented in this study are available within the article and supplementary data.

## Supplementary Materials

The following supporting information can be downloaded at: https://www.mdpi.com/article/10.3390/biomedicines10102433/s1, Figure S1: No hearing loss is detected in the STAT1-/- mouse surviving inoculation with 103 PFU of ML29 through 10wpi; Figure S2: ML29-inoculated STAT1-/- mice depleted of CD4 and CD8 T cells develop a systemic infection with persistent viremia through the end of the study, 82 dpi; Figure S3: STAT1-/- mice depleted of CD8 T cells succumbing to ML29 infection trend lower systemic viral titers than their CD4 T cell-depleted counterparts; Table S1: Viral dissemination of ML29 in STAT1-/- mice.

## References

[1] McCormick J.B., Fisher-Hoch S.P. (1972). Lassa fever. Br. Med. J..

[2] Centers for Disease Control and Prevention (2015). Lassa Fever. https://www.cdc.gov/vhf/lassa/index.html.

[3] Olayemi A., Oyeyiola A., Obadare A., Igbokwe J., Adesina A.S., Onwe F., Ukwaja K.N., Ajayi N.A., Rieger T., Gunther S. (2018). Widespread arenavirus occurrence and seroprevalence in small mammals, Nigeria. Parasit Vectors.

[4] Ibekwe T.S., Okokhere P.O., Asogun D., Blackie F.F., Nwegbu M.M., Wahab K.W., Omilabu S.A., Akpede G.O. (2011). Early-onset sensorineural hearing loss in Lassa fever. Eur. Arch. Otorhinolaryngol..

[5] Mustapha A. (2017). Lassa Fever: Unveiling the misery of the Nigerian health worker. Ann. Niger. Med..

[6] Walker D.H., McCormick J.B., Johnson K.M., Webb P.A., Konba-Kono G., Elliott L.H., Gardner J.J. (1982). Pathologic and virologic study of fatal Lassa fever in man. Am. J. Pathol..

[7] McCormick J.B., King I.J., Webb P.A., Scribner C.L., Craven R.B., Johnson K.M., Elliott L.H., Belmont-Williams R. (1986). Lassa fever. Effective therapy with ribavirin. N. Engl. J. Med..

[8] Ezeomah C., Adoga A.L., Ihekweazu C., Paessler S., Cisneros I., Tomori O., Walker D. (2019). Sequelae of Lassa Fever: Postviral Cerebellar Ataxia. Open Forum Infect. Dis..

[9] Li A.L., Grant D., Gbakie M., Kanneh L., Mustafa I., Bond N., Engel E., Schieffelin J., Vandy M.J., Yeh S. (2020). Ophthalmic manifestations and vision impairment in Lassa fever survivors. PLoS ONE.

[10] Mateer E.J., Huang C., Shehu N.Y., Paessler S. (2018). Lassa fever-induced sensorineural hearing loss: A neglected public health and social burden. PLoS Negl. Trop. Dis..

[11] Cummins D., McCormick J.B., Bennett D., Samba J.A., Farrar B., Machin S.J., Fisher-Hoch S.P. (1990). Acute sensorineural deafness in Lassa fever. JAMA.

[12] Dunmade A.D., Segun-Busari S., Olajide T.G., Ologe F.E. (2007). Profound bilateral sensorineural hearing loss in nigerian children: Any shift in etiology?. J. Deaf Stud. Deaf Educ..

[13] McElroy A.K., Akondy R.S., Harmon J.R., Ellebedy A.H., Cannon D., Klena J.d., Sidney J., Sette A., Mehta A.K., Kraft C.S. (2017). A Case of Human Lassa Virus Infection With Robust Acute T-Cell Activation and Long-Term Virus-Specific T-Cell Responses. J. Infect. Dis..

[14] ter Meulen J., Badusche M., Kuhnt K., Doetze A., Satoguina J., Marti T., Leoliger C., Koulemou K., Koivogui L., Schmitz H. (2000). Characterization of human CD4(+) T-cell clones recognizing conserved and variable epitopes of the Lassa virus nucleoprotein. J. Virol..

[15] Baize S., Marianneau P., Loth P., Reynard S., Journeaux A., Chevallier M., Tordo N., Deubel V., Contamin H. (2009). Early and strong immune responses are associated with control of viral replication and recovery in lassa virus-infected cynomolgus monkeys. J. Virol..

[16] Cashman K.A., Wilkinson E.R., Zeng X., Cardile A.P., Facemire P.R., Bell T.M., Bearss J.J., Shaia C.I., Schmaljohn C.S. (2018). Immune-Mediated Systemic Vasculitis as the Proposed Cause of Sudden-Onset Sensorineural Hearing Loss following Lassa Virus Exposure in Cynomolgus Macaques. mBio.

[17] Yun N.E., Ronca S., Tamura A., Koma T., Seregin A.V., Dineley K.T., Miller M., Cook R., Shimizu N., Walker A.G. (2015). Animal Model of Sensorineural Hearing Loss Associated with Lassa Virus Infection. J. Virol..

[18] Flatz L., Rieger T., Merkler D., Bergthaler A., Regen T., Schedensack M., Bestmann L., Verschoor A., Kreutzfeldt M., Bruck W. (2010). T cell-dependence of Lassa fever pathogenesis. PLoS Pathog..

[19] Maruyama J., Reyna R.A., Kishimoto-Urata M., Urata S., Manning J.T., Harsell N., Cook R., Huang C., Nikolich-Zugich J., Makishima T. (2022). CD4 T-cell depletion prevents Lassa fever associated hearing loss in the mouse model. PLoS Pathog..

[20] Lukashevich I.S., Patterson J., Carrion R., Moshkoff D., Ticer A., Zapata J., Brasky K., Geiger R., Hubbard G.B., Bryant J. (2005). A live attenuated vaccine for Lassa fever made by reassortment of Lassa and Mopeia viruses. J. Virol..

[21] Lukashevich I.S. (1992). Generation of reassortants between African arenaviruses. Virology.

[22] Johnson D.M., Jokinen J.D., Lukashevich I.S. (2019). Attenuated Replication of Lassa Virus Vaccine Candidate ML29 in STAT-1(-/-) Mice. Pathogens.

[23] Suzuki T., Maruyama J., Cook R., Urata S., Paessler S., Makishima T. (2021). Auditory function analysis in immunodeficient STAT1 knock-out mice: Considerations for viral infection models. Neurosci. Lett..

[24] Lukashevich I.S., Paessler S., de la Torre J.C. (2019). Lassa virus diversity and feasibility for universal prophylactic vaccine. F1000Research.

[25] Lukashevich I.S. (1985). Lassa virus lethality for inbred mice. Ann. Soc. Belg. Med. Trop..

[26] Lukashevich I.S. (2013). The search for animal models for Lassa fever vaccine development. Expert Rev. Vaccines.

[27] Goicochea M.A., Zapata J.C., Bryant J., Davis H., Salvato M.S., Lukashevich I.S. (2012). Evaluation of Lassa virus vaccine immunogenicity in a CBA/J-ML29 mouse model. Vaccine.

[28] Baltes A., Akpinar F., Inankur B., Yin J. (2017). Inhibition of infection spread by co-transmitted defective interfering particles. PLoS ONE.

[29] Lopez C.B. (2014). Defective viral genomes: Critical danger signals of viral infections. J. Virol..

[30] Rezelj V.V., Levi L.I., Vignuzzi M. (2018). The defective component of viral populations. Curr. Opin. Virol..

[31] Johnson D.M., Cubitt B., Pfeffer T.L., de la Torre J.C., Lukashevich I.S. (2021). Lassa Virus Vaccine Candidate ML29 Generates Truncated Viral RNAs Which Contribute to Interfering Activity and Attenuation. Viruses.

[32] Lukashevich I.S., Pushko P. (2016). Vaccine platforms to control Lassa fever. Expert Rev. Vaccines.

[33] Pannetier D., Faure C., Georges-Courbot M.C., Deubel V., Baize S. (2004). Human macrophages, but not dendritic cells, are activated and produce alpha/beta interferons in response to Mopeia virus infection. J. Virol..

[34] Pannetier D., Reynard S., Russier M., Journeaux A., Tordo N., Deubel V., Baize S. (2011). Human dendritic cells infected with the nonpathogenic Mopeia virus induce stronger T-cell responses than those infected with Lassa virus. J. Virol..

[35] Baize S., Pannetier D., Faure C., Marianneau P., Marendat I., Georges-Courbot M.C., Deubel V. (2006). Role of interferons in the control of Lassa virus replication in human dendritic cells and macrophages. Microbes Infect..

[36] Carnec X., Mateo M., Page A., Reynard S., Hortion J., Picard C., Yekwa E., Barrot L., Barron S., Vallve A. (2018). A Vaccine Platform against Arenaviruses Based on a Recombinant Hyperattenuated Mopeia Virus Expressing Heterologous Glycoproteins. J. Virol..

[37] Kiley M.P., Lange J.V., Johnson K.M. (1979). Protection of rhesus monkeys from Lassa virus by immunisation with closely related Arenavirus. Lancet.

[38] Walker D.H., Johnson K.M., Lange J.V., Gardner J.J., Kiley M.P., McCormick J.B. (1982). Experimental infection of rhesus monkeys with Lassa virus and a closely related arenavirus, Mozambique virus. J. Infect. Dis..

[39] Fehling S.K., Lennartz F., Strecker T. (2012). Multifunctional nature of the arenavirus RING finger protein Z. Viruses.

[40] Lukashevich I.S., Carrion R., Salvato M.S., Mansfield K., Brasky K., Zapata J., Cairo C., Goicochea M., Hoosien G.E., Ticer A. (2008). Safety, immunogenicity, and efficacy of the ML29 reassortant vaccine for Lassa fever in small non-human primates. Vaccine.

[41] Carrion R., Patterson J.L., Johnson C., Gonzales M., Moreira C.R., Ticer A., Brasky K., Hubbard G.B., Moshkoff D., Zapata J. (2007). A ML29 reassortant virus protects guinea pigs against a distantly related Nigerian strain of Lassa virus and can provide sterilizing immunity. Vaccine.

[42] Zapata J.C., Poonia B., Bryant J., Davis H., Ateh E., George L., Crasta O., Zhang Y., Slezak T., Jaing C. (2013). An attenuated Lassa vaccine in SIV-infected rhesus macaques does not persist or cause arenavirus disease but does elicit Lassa virus-specific immunity. Virol. J..

[43] Titov A., Kaminskiy Y., Ganeeva I., Zmievskaya E., Valiullina A., Rakhmatullina A., Petukhov A., Miftakhova R., Rizvanov A., Bulatov E. (2022). Knowns and Unknowns about CAR-T Cell Dysfunction. Cancers.

[44] Cohen B.E., Durstenfeld A., Roehm P.C. (2014). Viral causes of hearing loss: A review for hearing health professionals. Trends Hear..

[45] Haller T.J., Price M.S., Lindsay S.R., Hillas E., Seipp M., Firpo M.A., Park A.H. (2020). Effects of ganciclovir treatment in a murine model of cytomegalovirus-induced hearing loss. Laryngoscope.

[46] Schraff S.A., Schleiss M.R., Brown D.K., Meinzen-Derr J., Choi K.Y., Greinwalk J.H., Choo D.I. (2007). Macrophage inflammatory proteins in cytomegalovirus-related inner ear injury. Otolaryngol. Head Neck Surg..

[47] Bradford R.D., Yoo Y.G., Golemac M., Pugel E.P., Jonjic S., Britt W.J. (2015). Murine CMV-induced hearing loss is associated with inner ear inflammation and loss of spiral ganglia neurons. PLoS Pathog..

[48] Woolf N.K., Koehrn F.J., Harris J.P., Richman D.D. (1989). Congenital cytomegalovirus labyrinthitis and sensorineural hearing loss in guinea pigs. J. Infect. Dis..

[49] Park A.H., Gifford T., Schleiss M.R., Dahlstrom L., Chase S., McGill L., Li W., Alder S.C. (2010). Development of cytomegalovirus-mediated sensorineural hearing loss in a Guinea pig model. Arch. Otolaryngol. Head Neck Surg..

[50] Li L., Kosugi I., Han G.P., Kawasaki H., Arai Y., Takeshita T., Tsutsui Y. (2008). Induction of cytomegalovirus-infected labyrinthitis in newborn mice by lipopolysaccharide: A model for hearing loss in congenital CMV infection. Lab. Investig..

[51] Juanjuan C., Yan F., Li C., Haizhi L., Ling W., Xinrong W., Juan X., Tao L., Zongzhi Y., Suhua C. (2011). Murine model for congenital CMV infection and hearing impairment. Virol. J..

[52] Schachtele S.J., Mutnal M.B., Schleiss M.R., Lokensgard J.R. (2011). Cytomegalovirus-induced sensorineural hearing loss with persistent cochlear inflammation in neonatal mice. J. Neurovirol..

