# Supplementary material for: Depletion of CD4 and CD8 T Cells Reduces Acute Disease and Is Not Associated with Hearing Loss in ML29-Infected STAT1-/- Mice
Source: Biomedicines. 2022 Sep 29;10(10):2433. doi: 10.3390/biomedicines10102433 (PMC9598517; doi:10.3390/biomedicines10102433)
Supplement: Supplementary file 1 [file biomedicines-10-02433-s001.zip › biomedicines-1925713-supplementary.pdf]

SUPPLEMENTAL FIGURES AND TABLES

Table S1. Viral dissemination of ML29 in STAT1-/- mice. LD: lower than the limit of detection (<2.00). NT: not tested.

Animal ID	Inoculation dose	Sampling day (DPI)	Virus titer (log10 PFU/g)					Virus titer (log10 PFU/ml)
			Brain	Lung	Liver	Spleen	Kidney	Serum
M01	10^5 PFU	14	6.38	6.41	5.70	5.01	5.96	5.60
M02		16	6.69	6.32	5.48	6.00	6.00	4.00
M04		15	5.03	3.00	LD	LD	3.60	NT
M05		16	5.94	LD	3.30	LD	LD	LD
M06	10^4 PFU	10	LD	4.00	LD	2.88	LD	2.70
M07		10	LD	6.93	3.40	3.40	3.67	4.00
M08		15	5.91	5.67	4.96	3.78	5.48	4.95
M09		10	LD	5.00	2.91	LD	LD	2.30
M10		19	LD	LD	LD	LD	LD	LD
M11	10^3 PFU	70	LD	LD	LD	LD	LD	LD
M12		11	5.51	7.99	4.98	5.44	5.00	4.60
M13		11	LD	4.30	4.21	3.89	3.00	2.77
M14		13	3.00	6.12	3.41	LD	LD	3.48
M15		19	5.24	LD	LD	LD	5.00	LD
M16	10^2 PFU	13	2.81	6.20	LD	LD	3.00	4.00
M17		15	4.85	LD	LD	LD	LD	2.30
M18		13	2.76	6.72	4.49	LD	5.67	4.47
M19		14	LD	LD	LD	2.93	LD	LD
M21	10^1 PFU	14	4.45	6.68	3.13	3.83	4.48	3.48
M22		13	LD	5.60	LD	LD	4.34	2.00
M23		13	2.62	6.00	LD	3.22	LD	3.30
M24		13	4.61	4.00	LD	LD	3.00	3.00
M25		21	6.80	3.78	LD	3.60	6.08	2.30
M26	10^0 PFU	13	2.64	6.11	3.00	2.85	3.00	3.30
M27		12	5.99	8.70	6.00	6.23	5.85	4.48
M28		10	3.65	7.30	5.15	7.00	3.60	2.30
M29		11	4.19	8.60	6.10	5.34	5.48	4.00
M30		11	5.69	7.28	5.90	6.30	6.00	4.60

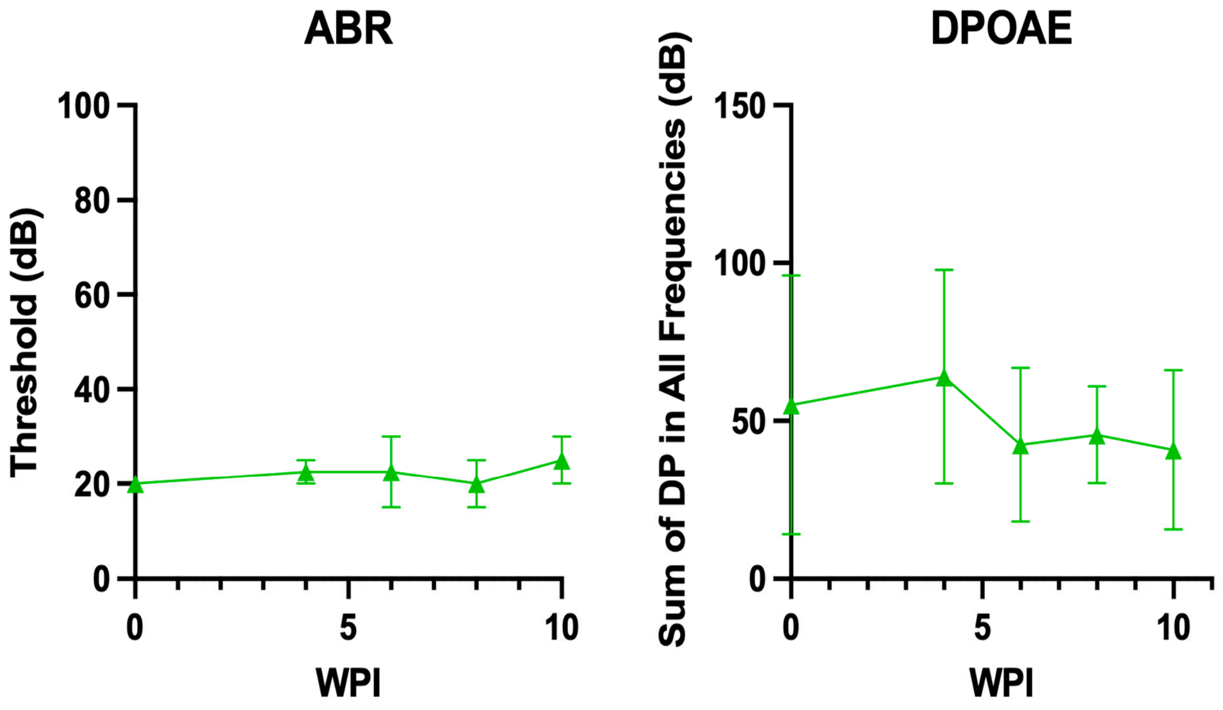

Supplemental Figure S1. No hearing loss is detected in the STAT1-/- mouse surviving inoculation with 10^3 PFU of ML29 through 10wpi. Data presented as average ± SEM. *n* = 2.

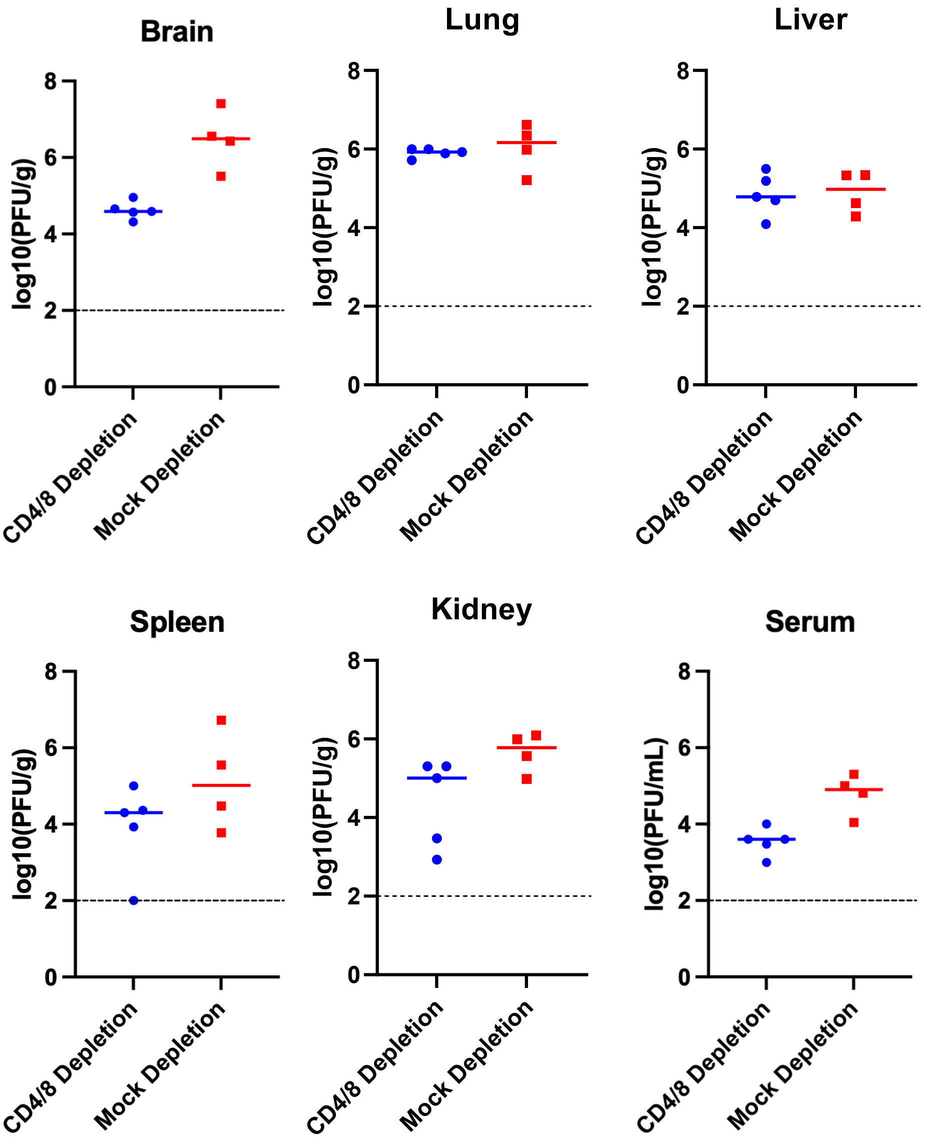

Supplemental Figure S2. ML29-inoculated STAT1-/- mice depleted of CD4 and CD8 T cells develop a systemic infection with persistent viremia through the end of the study, 82 dpi. On the contrary, mock depleted mice inoculated with ML29 develop a systemic infection. Data are plotted as individual values with the mean. 10^4, 10^3, 10^1, and 10^0 PFU n = 5; 10^5 and 10^2 PFU n = 4. All samples were collected at time of euthanasia, at 82dpi for CD4/CD8 T cell-depleted mice and between 13 and 17 dpi. Dashed line indicates lower limit of detection.

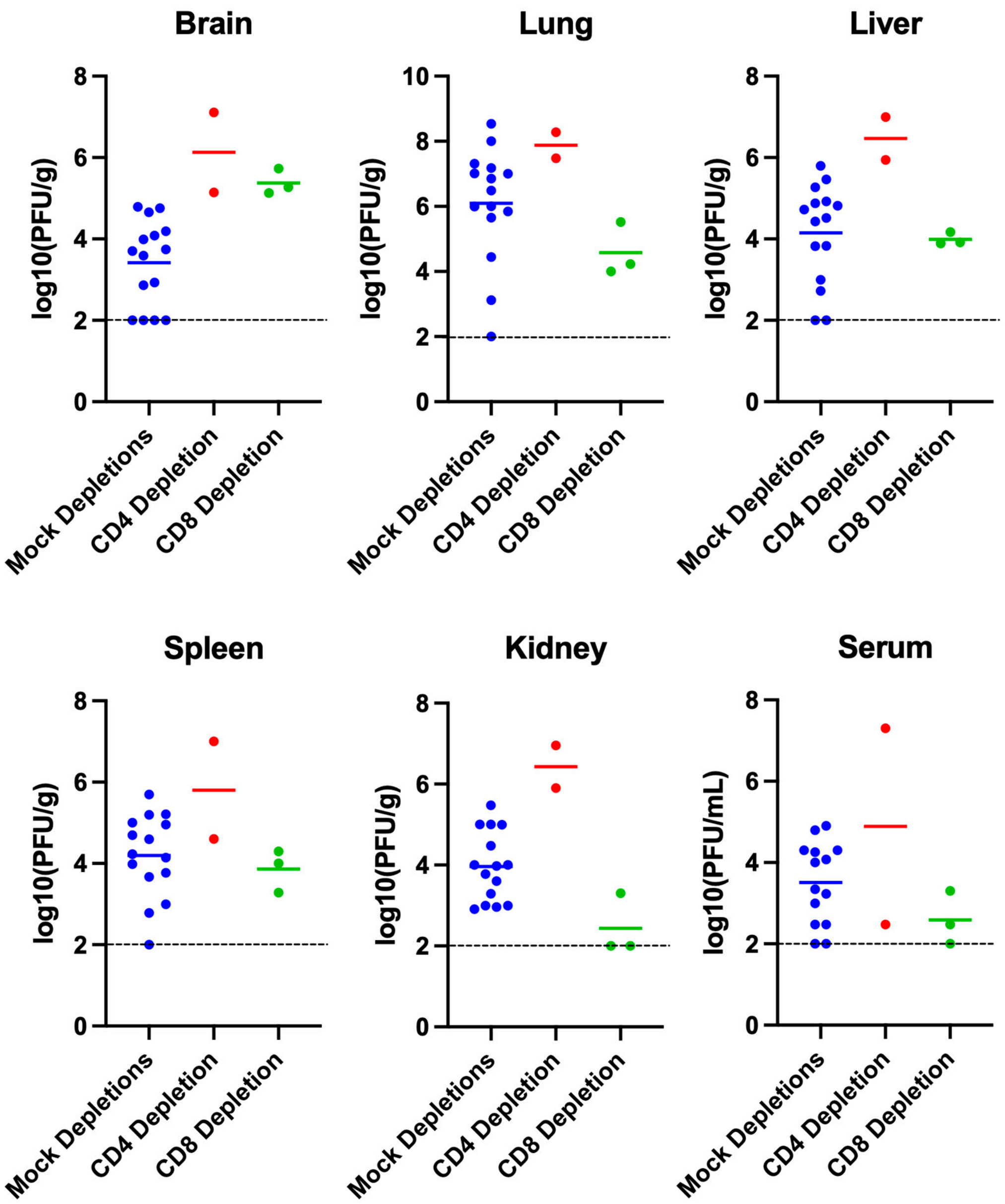

Supplemental Figure S3. STAT1-/- mice depleted of CD8 T cells succumbing to ML29 infection trend lower systemic viral titers than their CD4 T cell-depleted counterparts. Individual values plotted with mean. n = 14 (serum) n = 15 (organ) Mock Depletions; n = 3 CD8 Depletion; n = 2 CD4 Depletion. Dashed line indicates lower limit of detection.
